# A *Drosophila melanogaster* ortholog of *pentatricopeptide repeat domain 3* ( *PTCD3* ) is essential for development

**DOI:** 10.17912/micropub.biology.000999

**Published:** 2023-11-24

**Authors:** Eisuke Imura, Sora Enya, Ryusuke Niwa

**Affiliations:** 1 Graduate School of Life and Environmental Sciences, University of Tsukuba, Tsukuba, Ibaraki, Japan; 2 Life Science Center for Survival Dynamics, Tsukuba Advanced Research Alliance (TARA), University of Tsukuba, Tsukuba, Ibaraki, Japan

## Abstract

Mitochondrial DNA (mtDNA) replication and transcription are essential for cellular energy metabolism. It has been suggested that pentatricopeptide repeat (PPR) proteins regulate various aspects of mitochondrial RNA metabolism, including transcription, processing, maturation and stability, and protein synthesis. However, an *in vivo* requirement of PPR proteins in RNA metabolism has not been fully examined. In this paper, we focus on the *Drosophila melanogaster* homolog of *PPR domain 3* ( *PTCD3* ), encoded by the *CG4679* gene. A loss-of-function mutant of *PTCD3* is lethal during the second instar. In addition, mutants exhibit reduced expression of a group of genes related to mitochondrial function and ribosome biogenesis, and conversely, they show up-regulated expression of neuronal development-related genes. These results suggest that *PTCD3* has important functions in relation to mtDNA and is essential for development.

**Figure 1. Phenotypes of PTCD3 mutant animals:**
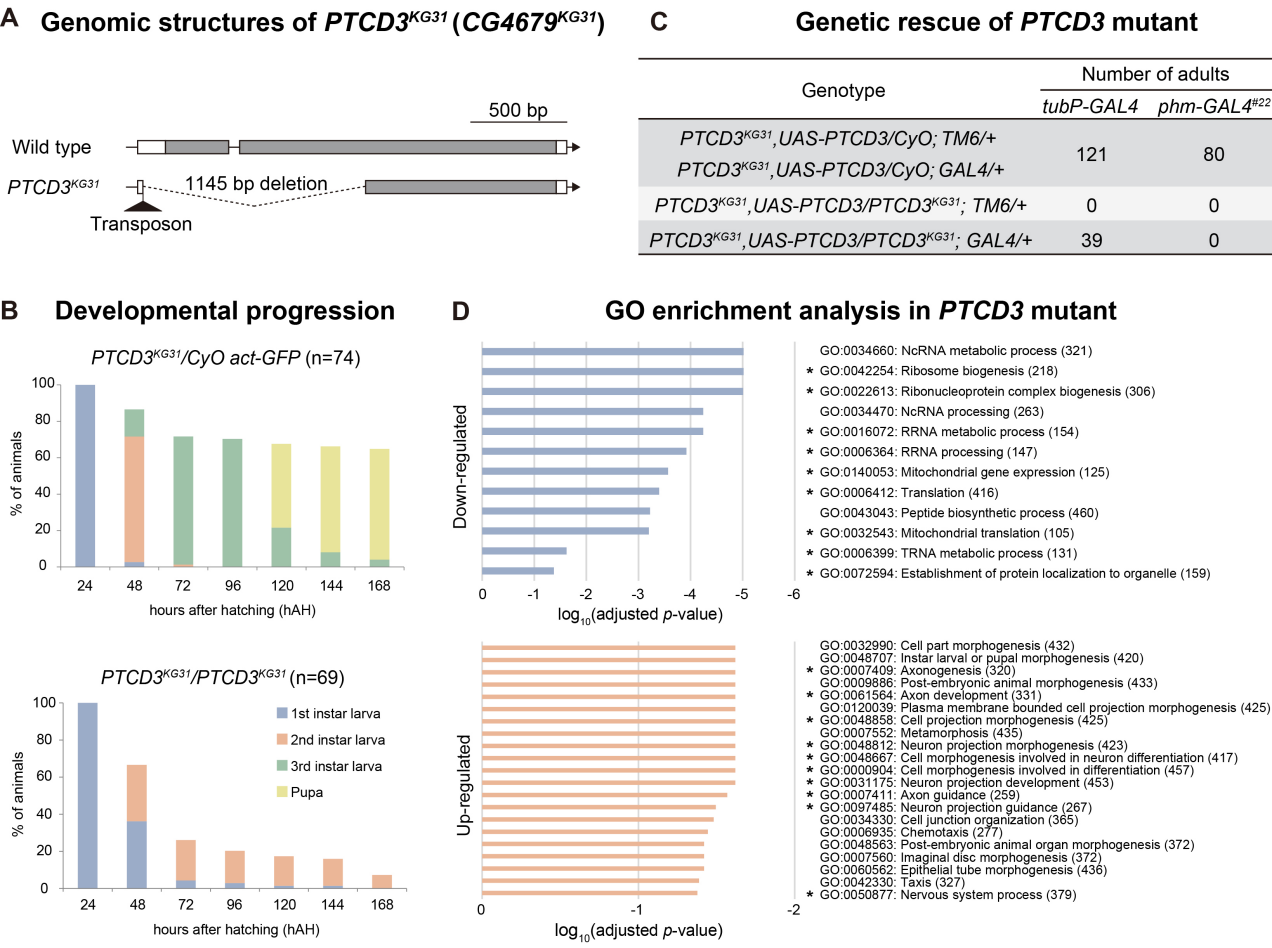
**(A)** **Genomic structure of *PTCD3 ^KG31^* .** Genomic structures of *PTCD3* ( *CG4679* ) loci of the wild type and knockout ( *PTCD3 ^KG31^* ) strains. Gray and white boxes indicate the coding sequence and untranslated regions, respectively. Arrows indicate orientations of the gene. In the *PTCD3 ^KG31^* allele, there is a 1145-bp deletion in the *PTCD3* gene region and a transposon is inserted there. **(B) Survival rate and developmental progression of *PTCD3* knockout mutants.** The survival rate and developmental progression of control ( *PTCD3 ^KG31^ /CyO act-GFP* , n=74, top panel) and *PTCD3* knockout mutants ( *PTCD3 ^KG31^ /PTCD3 ^KG3^* ^1^ , n=69, bottom panel). **(C) Genetic rescue of *PTCD3* knockout mutants by *PTCD3* overexpression in the whole organism and the prothoracic gland.** Numbers of *PTCD3 ^KG31^ /PTCD3 ^KG31^* animals that reached adulthood were scored. *PTCD3* was driven by *tubP-GAL4* and the *phm-GAL4 ^#22^* driver. **(D) Gene ontology enrichment analysis in *PTCD3* knockout mutants.** Significantly enriched gene ontology (GO) terms of down-regulated (top panel) and up-regulated genes (bottom panel) in *PTCD3* knockout mutants ( *PTCD3 ^KG31^ /PTCD3 ^KG3^* ^1^ ) compared to control ( *PTCD3 ^KG31^ /+* ) are listed in order along with adjusted *p* -values. Asterisks represent GO classifications related to ribosome biogenesis, mitochondrial gene expression, and neuronal development. Numbers in brackets indicate the total number of applicable genes. Ontology was set to biological process. All terms with adjusted *p* -values <0.05 (Fisher's exact test) and |fold change| >2 are displayed.

## Description

Mitochondrial DNA (mtDNA) replication and transcription are crucial to cellular energy metabolism. De-regulation of mitochondrial genome retention and gene expression disrupts cellular energy metabolism and is associated with various human diseases (Wallace 2005, Chen et al. 2019) . Mitochondrial RNA polymerase is a single subunit RNA polymerase responsible for mitochondrial transcription and contains a pentatricopeptide repeat (PPR) motif at its N terminus. PPR is a denatured motif consisting of 35 amino acids, frequently tandemly repeated (Schmitz-Linneweber and Small 2008) . PPR proteins are characterized as sequence-specific RNA-binding proteins involved in organellar transcription, RNA processing and stability, and translation (Rovira and Smith 2019, Manna 2015) . Mammals have only seven PPR domain proteins, all of which are located in mitochondria and which regulate various aspects of mitochondrial RNA metabolism, including transcription, processing, maturation and stability, and protein synthesis (Rackham and Filipovska 2012) . Nevertheless, an *in vivo* requirement of PPR proteins in RNA metabolism remains unclear.

*Drosophila melanogaster* shares with humans the same set of genes regulating mtDNA replication and transcription. Moreover, *Drosophila* and humans have similar general structure and organization of mtDNA (Gustafsson et al. 2016, Garesse 1988) . Therefore, *Drosophila* has been considered an attractive model for studying mtDNA maintenance and associated human diseases (Garesse and Kaguni 2005, Sánchez-Martínez et al. 2006). Recently, it was reported that the PPR domain of *Drosophila* mitochondrial RNA polymerase has exoribonuclease activity that is essential to synthesize short RNA oligonucleotides, so as to initiate DNA replication (Liu et al. 2022) . Here, we focused on another PPR protein, an ortholog of mammalian *PPR Domain 3 (PTCD3)* , encoded by the *CG4679* locus in *Drosophila* (FlyBase: https://flybase.org/reports/FBgn0033816.html FB2023_04) (Gramates et al. 2022) . In mammalian cells, PTCD3 binds to the small subunit of the mitochondrial ribosome and 12S rRNA. Knockdown and overexpression of *PTCD3* in 143B human osteosarcoma cells revealed that PTCD3 is not involved in RNA processing or stability, whereas it regulates mitochondrial protein translation and activity of complexes III and IV, modulating mitochondrial respiration (Davies et al. 2009, Lightowlers and Chrzanowska-Lightowlers 2013) .

Since the *in vivo* functional importance of *PTCD3* has not yet been examined in *Drosophila* , we generated a *PTCD3* loss-of-function allele using an imprecise P-element mobilization (See Methods for more details). We succeeded in isolating one mutant allele, *PTCD3 ^KG31^* , which had a large deletion in the N-terminal region, including the start codon of the coding region (Figure A).

Embryos homozygous for *PTCD3 ^KG31^* completed embryogenesis, hatched normally, and showed no obvious morphological defects after hatching. However, *PTCD3 ^KG31^* homozygotes arrested development during the first or second instar, and even ≥120 h after hatching (hAH), never molted into third instars (Figure B, bottom). Eventually, all *PTCD3 ^KG31^* homozygous animals died, retaining second instar morphology. In contrast, most control *PTCD3 ^KG31^ /CyO act-GFP* heterozygous animals became pupae (Figure B, top). These results suggest that *PTCD3* is necessary for larval development in *Drosophila* .

We next examined whether the larval arrest and lethality phenotype of *PTCD3 ^KG31^* mutants were certainly due to the loss of *PTCD3* . Ubiquitous overexpression of *PTCD3* using *tubP-GAL4* rescued the larval arrest phenotype of *PTCD3 ^KG31^* homozygotes, and some of the animals developed into adults (Figure C). This result demonstrates that the developmental arrest phenotype of *PTCD3 ^KG31^* mutant is due exclusively to loss of *PTCD3* function and that *PTCD3* is essential for *Drosophila* development.

In previous studies, a potential link between mitochondrial gene expression and the steroid biosynthetic pathway was suggested (Llorens et al. 2015, Jacobs et al. 2020) . In *Drosophila* , the insect steroid hormone, ecdysteroid, regulates onset of larval molting and pupariation (Kamiyama and Niwa 2022, Yamanaka 2021) . Since mammalian PTCD3 functions in mitochondrial gene expression and the *PTCD3 ^KG31^* mutant phenotype closely resembles the defective phenotype of ecdysteroid signaling-related genes, we assumed that *PTCD3* might be important in the prothoracic gland (PG), which is the site of ecdysteroid biosynthesis. However, overexpression of *PTCD3* in the PG using *phm-GAL4 ^#22^* did not rescue the larval arrest phenotype of *PTCD3 ^KG31^* homozygotes (Figure C).

Finally, we performed an RNA-sequencing analysis of *PTCD3 ^KG31^* mutants to determine whether *PTCD3* is involved in mitochondrial gene expression in *Drosophila* , as with mammalian PTCD3. We found down- and up-regulated genes associated with specific gene ontology (GO) terms in *PTCD3 ^KG31^* mutants, including ribosome biogenesis (Figure D, top) and neuronal development (Figure D, bottom). Importantly, genes classified as mitochondrial gene expression by GO enrichment analysis were significantly down-regulated genes in *PTCD3 ^KG31^* mutants, which is consistent with the function of *PTCD3* expected from mammalian cells.

In both mice and humans, *PTCD3* loss-of-function mutants exhibit defects in mitochondrial translational and mitochondrial respiratory systems, neurodevelopmental defects, and early lethality (International Mouse Phenotyping Consortium: https://www.mousephenotype.org/data/genes/MGI:1917206 Data release 19.1) (Groza et al. 2023, Borna et al. 2019) . In addition, human *PTCD3* is associated with Leigh syndrome or Leigh-like symptoms (Borna et al. 2019, Finsterer et al. 2019) . Given that the *Drosophila PTCD3* mutation is lethal in early larval stages and has defects in gene expression related to mitochondrial function and neuronal development, our data support the idea that the function of *Drosophila* PTCD3 is very similar to that of mammalian PTCD3. Thus, the *Drosophila PTCD3* mutant may potentially be used as a model for pathology of Leigh syndrome or Leigh-like symptoms.

## Methods

**Fly husbandry and stocks** Flies were raised on fly food (5.5 g agar, 100 g glucose, 40 g dry yeast, 90 g corn flour, 3 mL propionic acid, and 3.5 mL 10% butyl p-hydroxybenzoate (in 70% ethanol) per liter) in 12/12 h light/dark conditions at 25 °C. *w ^1118^* was used as the wild-type (control) strain. The *tubP-GAL4* (stock numbers #5138) strain was obtained from the Bloomington *Drosophila* Stock Center (BDSC) *. phm-GAL4 ^#22^* was a generous gift from Michael B. O'Connor (University of Minnesota, USA) (Ono et al. 2006) .

**Generation of *PTCD3 ^KG31^* allele** The loss-of-function strain *PTCD3 ^KG31^* was isolated by P-element imprecise excision as previously described (Robertson et al. 1988) . The fly strain *P{SUPor-P}CG4679KG09310* has a P-element insertion at the 5´ untranslated region of *CG4679* ( *PTCD3* ). The strain was crossed with *CyO Δ2-3* flies carrying a P-element transposase to induce remobilization of the P-element. We isogenized each P-element-excision line, obtained genomic DNA from each line, and then checked whether each line had a large deletion spanning the *PTCD3* coding region, using PCR with genomic DNA and the primers, CG4688jump-F2 and CG4688jump-R3. We eventually isolated *PTCD3 ^KG31^* , which has a 1,145 bp deletion spanning part of the 5´ untranslated region and the coding region of *PTCD3* (Figure A).

**Generation of a *UAS-PTCD3*** **transgenic strain**

To generate overexpression vectors of *PTCD3* , specific primers pENTER-CG4679-F and pENTER-CG4679stop-R were used for PCR with KOD Plus Neo (TOYOBO) to amplify the coding sequence (CDS) of *PTCD3* . Template cDNAs were reverse transcribed using total RNA of *D. melanogaster* Oregon R embryos (0 to 13 h after hatching) with Prime Script Reverse Transcriptase (Takara). The amplified CDS region of *PTCD3* was ligated into a pENTR/D-TOPO plasmid (Thermo Fisher Scientific). The pENTR/D-TOPO plasmid with *PTCD3* CDS was then subjected to Gateway cloning technology, by which the plasmid was mixed with a destination vector pWALIUM10-roe (DRSC/TRiP Functional Genomics Resources & DRSC-BTRR; RRID:DGRC_1471), a phiC31 integrase system-based *UAS* vector (Perkins et al. 2015) , and LR Clonase II Enzyme Mix (Thermo Fisher Scientific). The Gateway reaction was constructed in *UAS-PTCD3* . Transformants with the *UAS-PTCD3* /pWALIUM10-roe vector and a *attP40* strain (Markstein et al. 2008) were established by BestGene, Inc.

**Scoring of developmental progression of *PTCD3* mutants** Eggs were laid on grape plates with yeast pastes at 25°C for 4 h. First instar larvae were transferred into a single vial with standard cornmeal food (30 animals per vial). Vials were prepared at least 3 independently. Every 24 h, developmental stages were scored by tracheal morphology, as previously described (Niwa et al. 2010) .

**Genetic rescue experiments of *PTCD3* mutants by *PTCD3* overexpression** The fly line *PTCD3 ^KG31^ , UAS- PTCD3/CyO* was crossed with *PTCD3 ^KG31^ /CyO, tubP-GAL4/TM6* or *PTCD3 ^KG31^ /CyO, phm-GAL4 ^#22^ /TM6* . Eggs were laid on standard agar-cornmeal medium at 25°C for 24 h. After adults eclosed from all observed pupae, numbers of adults were scored based on the presence of *CyO* and *TM6* balancers.

**RNA-sequencing and gene ontology enrichment analysis** RNA sequencing was performed on *PTCD3 ^KG31^ /PTCD3 ^KG31^* and *PTCD3 ^KG31^ /+* at 24 hAH. Ten larvae were collected and homogenized in RNAiso Plus (9101, TaKaRa Bio, Kusatsu, Shiga, Japan) and frozen with liquid nitrogen. Three biological replicates in each genotype were analyzed. RNA extraction, RNA qualification, library preparation, and RNA-sequencing were performed by Tsukuba i-Laboratory, Inc. Reads were aligned to the *Drosophila melanogaster* genome (BDGP6.28)with HISAT2 2.2.1 (Cunningham et al. 2022, Kim et al. 2019) . Then, aligned reads at each gene locus were counted with Samtools 1.10 and Subread 2.0.1 (Li et al. 2009, Liao et al. 2014) . Gene ontology (GO) enrichment analysis was performed on differentially expressed genes by DESeq2 packages using iDEP 0.92 ( http://bioinformatics.sdstate.edu/idep92/ ) (Ge et al. 2018) . *p* -values were adjusted with the Benjamini–Hochberg false discovery rate (FDR). RNA-sequencing transcriptional data are available from the DNA Data Bank of Japan Sequence Read Archive (Accession number DRA016983).

## Reagents

**Table:** 

***Drosophila melanogaster* STRAIN **	**GENOTYPE**	**AVAILABLE FROM**
14982	*P{SUPor-P}CG4679KG09310*	BDSC
8201	*CyO, PBac{Δ2-3.Exel}2/amosTft*	BDSC
5138	*tubP-GAL4*	BDSC
* PTCD3 ^KG31^*	* PTCD3 ^KG31^*	This study
*UAS-PTCD3*	*P{UAS-PTCD3}attP40*	This study
* phm-GAL4 ^#22^*	* phm-GAL4 ^#22^*	M. B. O'Connor (University of Minnesota, USA)

**Table:** 

**PLASMID**	**GENOTYPE**	**DESCRIPTION**
*UAS-PTCD3* / pWALIUM10-roe	*UAS-PTCD3* / pWALIUM10-roe	The pWALIUM10-roe plasmid carrying the *PTCD3* wild-type CDS, whose expression is under control of *UAS* promoter.

**Table:** 

**PRIMER**	**SEQUENCE**
CG4688jump-F2	5´-CCCGTGGTTAGCCTGATGCTG-3´
CG4688jump-R3	5´-CGAACGCCTTGAAAGTCGATCAG-3´
pENTER-CG4679-F	5´-CACCATGTACCTCTCGCGCCAATTGAG-3´
pENTER-CG4679stop-R	5´-CTACTTATCGAGGAAACTTTCGCCTACGAG-3´
